# Thyromegaly: Not The Usual Cause

**DOI:** 10.7759/cureus.37750

**Published:** 2023-04-18

**Authors:** N.S. Thillai Arunachalam, Saranya N, Nagarajan Priyathersini, Arthi M, Suhaildeen Kajamohideen

**Affiliations:** 1 Pathology, Sri Ramachandra Institute of Higher Education and Research, Chennai, IND; 2 Surgical Oncology, Sri Ramachandra Institute of Higher Education and Research, Chennai, IND

**Keywords:** non-hodgkin lymphoma of thyroid, diffuse large b-cell lymphoma, thyroid swelling, immunohistochemistry, thyroid malignancies

## Abstract

Non-Hodgkin lymphoma (NHL) of the thyroid is a very rare condition. Swelling of the neck is the usual presentation among patients. Of all thyroid malignancies, only a very small portion accounts for NHL of the thyroid. Here, we present two cases of diffuse large B-cell NHL of the thyroid. Preoperative diagnosis helps in the management of patients with chemotherapy, but in rare cases, surgical removal of the thyroid is done to reduce obstructive symptoms. The diagnosis is usually made based on fine-needle aspiration cytology and biopsy with immunohistochemistry. In these two cases, the patients presented with a history of a rapidly growing mass in the neck for three to four months, but the treatment modalities differed. In one of the cases, the patient received six cycles of chemotherapy, and in the other case, the patient underwent a total thyroidectomy, followed by six cycles of chemotherapy, though chemotherapy is the standard treatment option over surgical removal of the thyroid.

## Introduction

Malignant melanoma is the proliferative neoplastic action of the lymphopoietic moiety of the reticuloendothelial system entailing cells of either histiocytic or lymphocytic series in discrete degrees of differentiation that occur in an essentially homogenous population of single cell type [1]. Primary thyroid lymphomas are very rare. It accounts for 2% of total thyroid malignancies [2]. The prevalence is also low which a ballpark figure of 2.1 per million persons [3].

Only 1.2-1.7% of all non-Hodgkin lymphomas (NHLs) are thyroid lymphomas [4]. The two cases presented here involve males but females have a greater predilection for developing thyroid lymphomas (4:1) [5]. It is important to distinguish between anaplastic carcinoma and lymphoma of the thyroid as a rapid increase in the size of pre-existing thyroid is a hallmark of both [6]. Improved diagnosis is given with the help of immunohistochemical markers [7]. Preoperative diagnosis allows therapeutic management by chemotherapy, but in some cases, surgical removal of the thyroid is done to reduce obstructive symptoms [8].

## Case presentation

Case one

A 63-year-old male patient presented with the chief complaints of neck swelling for four months with a rapid increase in size. He had difficulty swallowing. There was no dyspnea. He did not complain of any weight loss or night sweats. The patient had been diagnosed with type 2 diabetes mellitus and hypothyroidism and was on treatment. The patient had normal bladder and bowel habits with normal sleep and appetite. There was no other organomegaly.

On general examination the Eastern Cooperative Oncology Group (ECOG) score was 1, the pulse rate was 80 beats per minute, and blood pressure was 120/80 mmHg. On general examination, pallor, icterus, cyanosis, clubbing, and pedal edema were absent. On cardiovascular examination, S1 and S2 were heard with no associated murmurs. Normal vesicular breath sounds were heard on respiratory system examination. Bilateral air entry was present.

**Figure 1:**
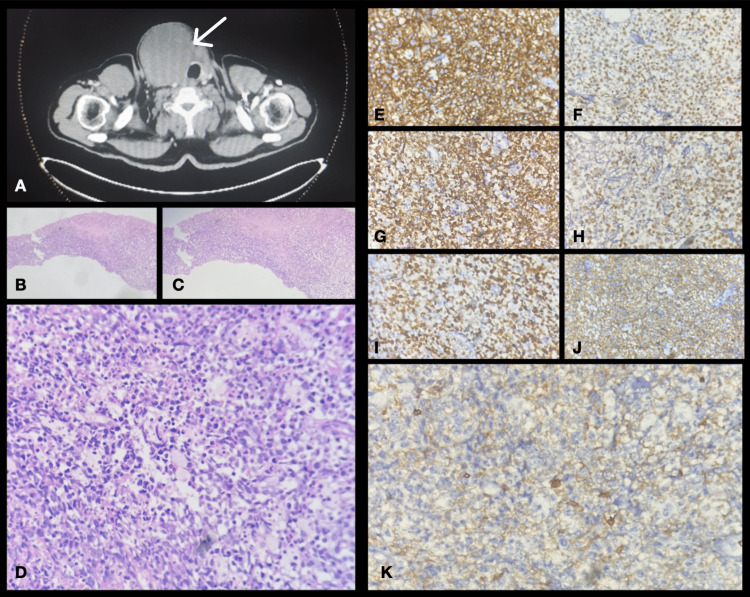
Case one. A: CT image showing lesions in the thyroid gland bilaterally. B: Fragments of the thyroid tissue with diffuse infiltrates of atypical lymphoid cells (H&E, 50×). C: Fragments of the thyroid tissue with diffuse infiltrates of atypical lymphoid cells (H&E, 100×). D: Diffuse infiltrates of atypical lymphoid cells with numerous mitosis and apoptosis (H&E, 400×). E: Diffuse membranous positivity of CD45 (IHC, 400×). F: Diffuse nuclear positivity of MUM1 (IHC, 400×). G: Diffuse nuclear and cytoplasmic positivity of BCL2 (IHC, 400×). H: Diffuse nuclear positivity of BCL6 (IHC, 400×). I: Diffuse nuclear positivity of Ki67 of 90% (IHC, 400×). J: Diffuse membranous positivity of CD20 (IHC, 400×). K: Scattered CD5 strong membranous positivity in the surrounding reactive T cells (IHC, 400×). H&E: hematoxylin & eosin; IHC: immunohistochemistry

There was a 20 × 20 cm swelling on the right side of the patient’s neck. Ultrasound-guided biopsy of the right neck lesion was done (Figure 1, Panel A). During the procedure, the patient went into bradycardia and was shifted to the intensive care unit (ICU) and treated symptomatically. The patient’s pulse rate improved. Re biopsy of the lesion was attempted and the specimens were sent to the pathology department.

The pathology department received five linear cores of gray-white soft-tissue fragments, with the largest measuring 0.6 × 0.2 × 0.1 cm and the smallest measuring 0.4 × 0.2× 0.1 cm. Microscopy showed linear cores of tissue with diffuse infiltration of the tumor with large hyperchromatic pleomorphic nuclei with scanty to moderate cytoplasm (Figure 1, Panels B, C). Numerous atypical mitosis, apoptotic bodies, and areas of necrosis were seen (Figure 1, Panel D).

In addition, CD45 was diffusely positive (Figure 1, Panel E), CD3 was positive in reactive T cells, CD20 was diffusely positive (Figure 1, Panel J), CD10 was negative, BCL6 was positive (Figure 1, Panel H), MUM1 was positive (Figure 1, Panel F), BCL2 was positive (Figure 1, Panel G), cyclin D1 was negative, CD5 was positive in reactive T cells (Figure 1, Panel K), Ki67 was 90% (Figure 1, Panel I), and C-MYC was positive. These immunohistochemistry patterns were in favor of NHL of the thyroid, diffuse large B-cell lymphoma of the activated B-cell type.

The first cycle of chemotherapy was planned and was started with an injection of rituximab 600 mg in 500 mL normal saline (NS) over four hours, an injection of adriamycin 90 mg in 100 mL NS over 10 minutes, an injection of cyclophosphamide 1,400 mg in 500 mL NS over one hour, an injection of vincristine 2 mg IV bolus, and a tablet of prednisolone 100 mg OD for five days. The patient was stable post-chemotherapy and was discharged. Five more cycles of chemotherapy were done, and the patient recovered completely.

Case two

A 64-year-old male presented with complaints of bleeding and hoarseness of voice. He had complained of swelling in the front of the neck for three months. The patient was normal three months earlier, after which he developed swelling in front of the neck which was rapidly increasing in size. There was no history of dysphagia, pain, loss of weight, or loss of appetite. There was no history of hypo or hyperthyroidism. He was a known case of hypertension and was on medication for 10 years. He had normal bladder and bowel habits with normal sleep and appetite.

On general examination, the pulse rate was 82 beats per minute, respiratory rate was 20 breaths per minute, and blood pressure was 140/90 mmHg. There was no pallor, icterus, cyanosis, clubbing, or pedal edema. On cardiovascular examination, S1 and S2 were heard and no murmurs were associated. Normal vesicular breathing sounds were heard on respiratory system examination. Bilateral air entry was present.

There was a 7 × 7 cm swelling in front of the neck which involved both the lobes of the thyroid. The swelling moved with deglutition. The swelling had a bosselated surface, firm to hard in consistency, with the trachea in the midline. A total thyroidectomy was done with Kocher’s incision. Post-surgery, all baseline investigations were done and were within normal limits. After achieving anesthetic fitness, the patient was shifted to the ICU because of a variation in heart rate. A cardiologist’s opinion was taken who gave some medication following which the patient improved.

**Figure 2:**
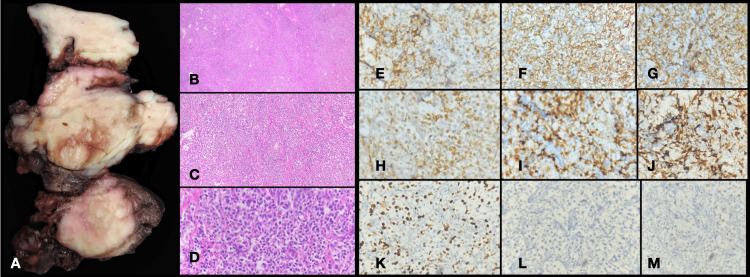
Case two. A: Total thyroidectomy specimen showing diffuse tan-brown solid lesion. B: Fragments of the thyroid tissue with diffuse infiltrates of atypical lymphoid cells (H&E, 50×). C: Fragments of the thyroid tissue with diffuse infiltrates of atypical lymphoid cells (H&E, 100×). D: Diffuse infiltrates of atypical lymphoid cells with scattered mitosis and apoptosis (H&E, 400×). E: Diffuse membranous positivity of CD5 (IHC, 400×). F: Diffuse membranous positivity of CD45 (IHC, 400×). G: Diffuse membranous positivity of CD20 (IHC, 400×). H: Diffuse nuclear and cytoplasmic positivity of BCL2 (IHC, 400×). I: Diffuse nuclear positivity of MUM1 (IHC, 400×). J: Diffuse nuclear positivity of BCL6 (IHC, 400×). K: Diffuse nuclear positivity of Ki67 of 70% (IHC, 400×). L: CD3 negative in tumor cells (IHC, 400×). M: Scattered nuclear positivity of C-MYC of 20% (hence negative) (IHC, 400×). H&E: hematoxylin & eosin; IHC: immunohistochemistry

The removed thyroid was sent to the pathology department. The total thyroidectomy specimen weighed 153 g, with the right lobe measuring 5 × 4 × 2.5 cm, the left lobe measuring 7 × 4 × 3 cm, and the isthmus measuring 8 × 6 × 3 cm. The external surface was bosselated and gray-brown, and the cut surfaces were gray-white, diffuse, firm, and homogenous (Figure 2, Panel A). Microscopy showed diffuse large B-cell lymphoma, with the adjacent thyroid parenchyma showing lymphocytic thyroiditis, and infiltration of tumor cells seen into the adjacent fatty tissue and muscle tissue (Figure 2, Panels B-D).

Four lymph nodes examined showed atypical lymphoid proliferation favoring lymphoma. In addition, CD45 was positive (Figure 2, Panel F). CD5 (Figure 2, Panel E) and CD20 were positive in neoplastic cells (Figure 2, Panel G), CD10 was negative, BCL2 was positive (Figure 2, Panel H), BCL6 was positive (Figure 2, Panel J), cyclin D1 was negative, MUM1 was positive (Figure 2, Panel I), C-MYC was negative (Figure 2, Panel M), and Ki67 was 70% (Figure 2, Panel K). This immunophenotyping was suggestive of NHL, diffuse large B-cell lymphoma. The patient was advised to undergo a cycle of chemotherapy.

## Discussion

The malignancies of the lymphoid system account for about 8% of all malignancies which are further categorized as Hodgkin disease and NHL. In the past two decades, there is an increase in the incidence of extranodal lymphomas [9]. Primary lymphomas of the thyroid are common among middle-aged women [10]. Clinically, NHL can mimic anaplastic thyroid carcinoma [11]. They usually present as a growing mass in the neck in the anterior aspect [12]. It might be associated with obstructive symptoms, and cervical lymphadenopathy is usually seen in these patients.

Studies suggest that autoimmune thyroiditis leads to the development of lymphomas [5]. B-cell lymphomas and large-cell lymphomas are usual among thyroid lymphomas [13]. Most common primary thyroid lymphomas include marginal-zone B lymphomas or diffuse large B-cell lymphomas. Diffuse large B-cell lymphoma is the most common type of primary thyroid malignancy and the second most common type, followed by the mucosa-associated lymphoid tissue type [14]. Lobectomy was earlier used as a diagnostic modality followed by immunohistochemistry analysis.

Diffuse large B-cell lymphoma has diverse molecular backgrounds of intermediate and high B-cell lymphomas which mostly spread to extranodal organs [15]. There are more than two extranodal sites involved commonly. Patients will less often have hyperthyroidism but may have hypothyroidism commonly [16,17]. Investigation of lymphadenopathy is through fine-needle aspiration cytology which is also cost-efficient compared to a surgical biopsy [18]. It is used as a preoperative diagnosis modality. Chemotherapy combined with radiotherapy is generally followed for the management of patients, but surgery becomes necessary if there is a critical airway obstruction.

## Conclusions

The most common presenting features of NHL of the thyroid include a mass in the thyroid gland, dysphagia, and dyspnea. Aggressive lymphomas are treated with a combined modality of chemotherapy and radiotherapy, whereas lymphomas with indolent histology are treated with radiotherapy. All treatment-related outcomes of thyroid lymphoma can be overcome by the combined treatment modality. When the diagnosis is uncertain or if there are obstructive symptoms, surgery becomes necessary.
